# A Biologically Inspired Network Design Model

**DOI:** 10.1038/srep10794

**Published:** 2015-06-04

**Authors:** Xiaoge Zhang, Andrew Adamatzky, Felix T.S. Chan, Yong Deng, Hai Yang, Xin-She Yang, Michail-Antisthenis I. Tsompanas, Georgios Ch. Sirakoulis, Sankaran Mahadevan

**Affiliations:** 1 School of Computer and Information Science, Southwest University, Chongqing 400715, China; 2 Unconventional Computing Center, University of the West of England, Bristol BS16 1QY, UK; 3 Department of Industrial and Systems Engineering, The Hong Kong Polytechnic University, Hung Hum, Kowloon, Hong Kong; 4 School of Engineering, Vanderbilt University, Nashiville, 37235, USA; 5 Department of Civil and Environmental Engineering, The Hong Kong University of Science and Technology, Clear Water Bay, Kowloon, Hong Kong; 6 School of Science and Technology, Middlesex University, London NW4 4BT, UK; 7 Department of Electrical and Computer Engineering, Democritus University of Thrace, Xanthi 67100, Greece

## Abstract

A network design problem is to select a subset of links in a transport network that satisfy passengers or cargo transportation demands while minimizing the overall costs of the transportation. We propose a mathematical model of the foraging behaviour of slime mould *P. polycephalum* to solve the network design problem and construct optimal transport networks. In our algorithm, a traffic flow between any two cities is estimated using a gravity model. The flow is imitated by the model of the slime mould. The algorithm model converges to a steady state, which represents a solution of the problem. We validate our approach on examples of major transport networks in Mexico and China. By comparing networks developed in our approach with the man-made highways, networks developed by the slime mould, and a cellular automata model inspired by slime mould, we demonstrate the flexibility and efficiency of our approach.

Transport networks are vital infrastructure of human society. Many networks are overloaded and often choked with traffic. Governments in most countries aim to ease congestion by imposing pollution charges^1^,^2^, tradable travel credits for congestion management^3^, parking permits distribution and trading^4^. Such policies reduce the congestion to some extent, but they also lead to additional problems. For example, in the policy of tradable travel credits for congestion management, it might be complicated to make a decision on how to allocate the limited tradable travel credits to users, how many tradable travel credits should be provided, etc^5^,^6^,^7^. One of the most promising solutions to this problem would be to design an efficient transit network for a transportation system. The efficient transit network is capable of maximizing throughout capacity and minimizing the overall costs^8^,^9^,^10^,^11^,^12^,^13^,^14^.

The network design problem (NDP) is one of the most challenging transport problems. The problem is defined as follows. Given a weighted graph *G*, we want to select such a subgraph in *G* that it satisfies the given point-to-point demand on a transportation and minimizes the overall costs of the transportation. In the past decades, various approaches have been presented to address this issue. The solutions can be divided into two categories: exact solutions^15^,^16^,^17^ and heuristic solutions^18^,^19^,^20^,^21^. Exact solution methods can deal with NDP in a rigorous manner. However, they are inefficient when dealing with large-scale real-world networks^18^,^22^,^23^. Heuristic approaches, emerged in the past decades^24^,^25^, provide approximate yet efficient solutions. The heuristic approaches can tackle a real-world problems with a large number of design variables^26^,^27^ and therefore these approaches are more popular than exact solutions^20^,^28^,^29^,^30^,^31^.

When we design a transportation network we should, ideally, make it fault tolerant, capable to cope with traffic accidents, terrorist attack, and emergency road maintenance. Fault tolerance and high performance attract higher building and maintenance costs. How to make a tradeoff between the overall cost, the fault tolerance, and the performance is the problem worthwhile to investigate^32^,^33^,^34^. During their evolution living creatures optimised their transportation networks over million of years: vascular systems of plants and animals^35^,^36^, foraging patterns of social insects^37^,^38^ migration trails by birds and animals, hunting routes of predators. It is therefore often very fruitful to apply natural solutions in designs of human-made artifacts^39^.

There is a unique creature which exhibits properties of internal and external living transport systems. This is a acellular slime mould *Physarum polycephalum*. Plasmodium is a vegetative stage of acellular slime mould *P. polycephalum*, a syncytium, a single cell with many nuclei, which feeds on microscopic particles^40^. When foraging for its food the plasmodium propagates towards sources of food particles, surrounds them, secretes enzymes and digests the food. Typically, the plasmodium forms a congregation of protoplasm covering the food source. When several sources of nutrients are scattered in the plasmodium’s range, the plasmodium forms a network of protoplasmic tubes connecting the masses of protoplasm at the food sources.

During its foraging behaviour the plasmodium spans scattered sources of nutrients with a network of protoplasmic tubes. The protoplasmic network is optimized to cover all sources of food and to provide a robust and speedy transportation of nutrients and metabolites in the plasmodium body. The plasmodium’s foraging behaviour can be interpreted as a computation, where data are represented by spatial configurations of attractants and repellents, and results of the computation are protoplasmic network formed by the plasmodium on the data sets^41^,^42^,^43^. The problems solved by plasmodium of *P. polycephalum* include the shortest path^41^,^42^, connecting different arrays of food sources in an efficient manner^44^, implementation of storage modification machines^45^, Voronoi diagram^46^, Delaunay triangulation^43^, logical computing^47^, and process algebra^48^, see overview in^43^.

A mathematical model of *Physarum* morphological behavior was proposed in^49^. Bonifaci *et al.*^50^ demonstrated that *Physarum* converges to a shortest path in the network regardless of the initial structure of the network or of the initial mass distribution. In the present paper, we explore *Physarum* to solve an NDP in terms of costs, efficiency and robustness. We employ the gravity model^51^ to estimate the traffic flow between a pair of cities. Based on a specific travel demand, we employ *Physarum* to simulate the transport flow between the cities. Then we allow the slime mould to colonise all cities, develop its protoplasmic network and settle down in some stable sate. The stable state represents a solution of the NDP.

## Results

We will evaluate the networks using measures of cost, efficiency, and robustness. In *Physaurm* algorithm, a threshold value *δ* as shown in the supplementary material is required to stop the execution of this program. If the threshold value *δ* is too small, it will take the *Physarum* algorithm a lot of time to converge to a solution. If *δ* is too large, the results will not reflect the features of the formulated networks. Here, we adopt a comprising strategy between the execution time of the algorithm and the characteristics of the formulated networks. The threshold value *δ* is set to be 0.01.

A cost (TL) is the sum of the length of all the edges existing in each network while the length is a representative of geographical distance. We have normalized the cost TL to the total length of the Minimum Spanning Tree (MST) for the corresponding networks. Efficiency (MD) is the transportation performance of each network, which is measured as the sum of minimum distance (MD) between all pairs of nodes. The efficiency MD is also normalized to the sum *MD*_*MST*_ of minimum distances between all pairs of nodes in the Minimum Spanning Tree. Finally, the fault tolerance, or robustness, of a network is measured as the probability of the network to become disconnected when a single link is removed. Here, the disconnection is defined as follows: for any pair of nodes, if there is no feasible path between them, we can say the network is disconnected. For example, in MST, the removal of any link will lead to the disconnection of the network.

### Application to Mexican Highways Network

**Figure 1:**
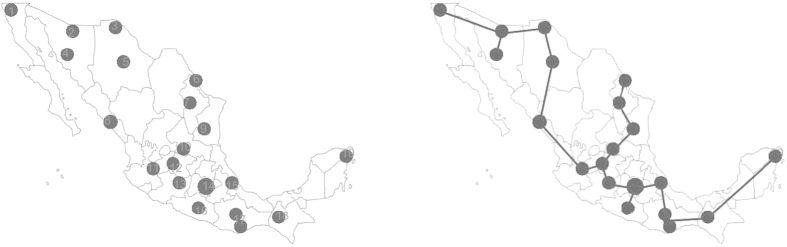
Basis of experiments with Mexico. (**a**) Configuration of sources of food representing major urban areas. (**b**) Minimum spanning tree. The maps are generated using the locations of these cities and the boundaries of Mexico. Maps created by AA.

To compare our algorithm with the real slime mould, we have used the results from our previous experimental laboratory studies^52^. We have selected 19 most populated urban areas shown in Fig. 1(a). The general data on these cities are described in Table 1. Figure 1(b) shows the minimum spanning tree (MST) of Mexico highways.

According to Eq. (8) and the data shown in Table 1, we can construct the basic traffic flow among any two cities. For the row with more than one economic power shown in Table 1, we can regard it as a single row by summing these economic powers, then the corresponding traffic flow can be determined. Specifically speaking, for Row 14, the economic power will be 2 + 1 + 7 = 10. According to Eq. (8), we can figure out the traffic flows from this city to the other cities.

**Figure 2:**
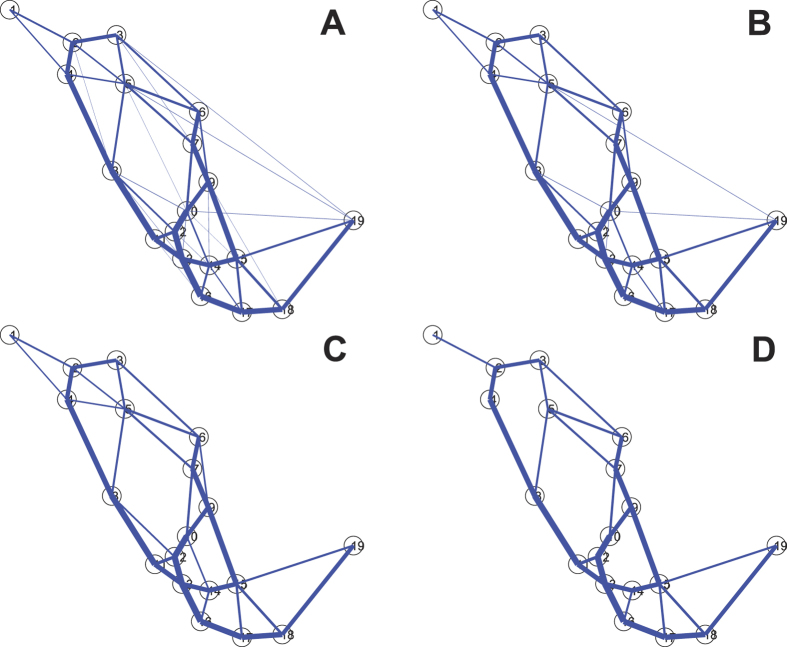
Comparison of the networks constructed by the *Physarum* when *α* has different values. (**A**) The network constructed by *Physarum* when the edges with conductivity less than 0.01 are filtered out. (**B**) The network with edges with *α* less than 0.05 are eliminated. (**C**) The constructed network when the edges with *α* less than 0.16, are eliminated. (**D**) The generated network when *α* is 0.26. (Here, a cost (TL) is the sum of the length of all the edges existing in each network while the length is a representative of geographical distance. We have normalized the cost TL to the total length of the Minimum Spanning Tree (MST) for the corresponding networks. An efficiency (MD) is the transportation performance of each network, which is measured as the sum of minimum distance (MD) between all pairs of nodes. The efficiency MD is normalized to the sum *MD*_*MST*_ of minimum distances between all pairs of nodes in the Minimum Spanning Tree. Finally, the fault tolerance, or robustness, of a network is measured as the probability of the network to become disconnected when a single link is removed).

The final step is to construct the network according to the conductivity value *α* associated with each edge. *α* is just used to filter out the edges with conductivity less than *α*. *α*'s values are determined as follows. When the *Physarum* algorithm is over, we will make full use of this parameter to build the networks with different topologies. Every time, these three parameters (Cost, Performance, and Efficiency) change, *α* will be recorded. We keep recording *α*'s values until the network becomes disconnected. Generally speaking, *α*'s values can reflect the changing trend of these formulated networks. With the increase of *α*, the less important edges will gradually fade out, which will affect the performance of the formulated network further. In this paper, we set the starting value for *α* is 0.01. For example, Fig. 2 shows four networks generated by *Physarum* when *α* is 0.01, 0.05, 0.16, 0.26, respectively.

**Figure 3:**
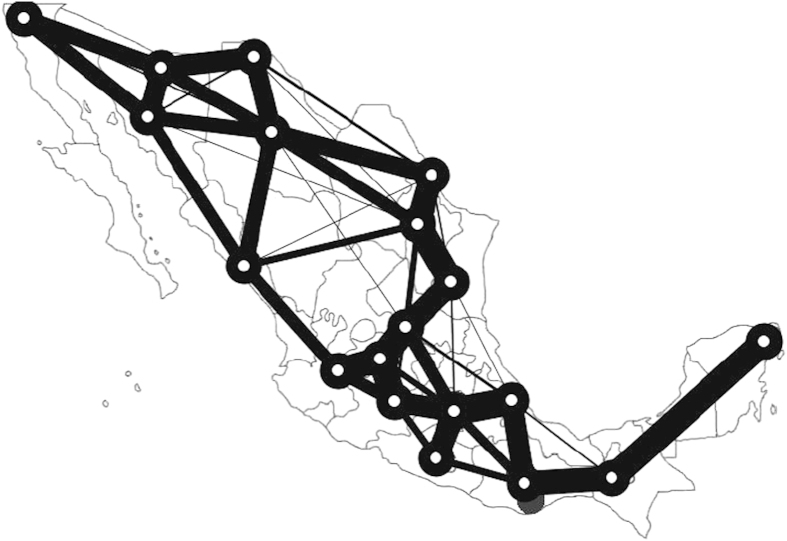
Mexico transport networks developed by the slime mould^52^. The maps are generated using the experimental results developed by the slime mould. Maps created by AA.

Let us now compare the transport performance, fault tolerance, and efficiency of each network. The real Mexico highways and the network developed by *Physarum* are shown in Fig. 3. ue to the reason of the copyright, we cannot show the structure of the real Mexico highways here. But it can be obtained from Ref. 52, which is Fig. 7(a) in Ref. 52. We also compare our results with a cellular automata model, inspired by slime mould, proposed by Tsompanas *et al.*^53^. The cellular automata model employs an attractant diffusion equation to describe the foraging behavior of the plasmodium and to calculate the propagation of chemoattractants produced by the nutrient sources. The diffusion of chemoattractants is uniform, while the growth of the slime mould is affected by the concentration of chemoattractants^53^,^54^. Note here that the parameters of the model are the same as used in^54^. Moreover, the input data of the model is only the configuration of the cities in the country and its borders. No economical or population factors are taken into consideration.

**Figure 4:**
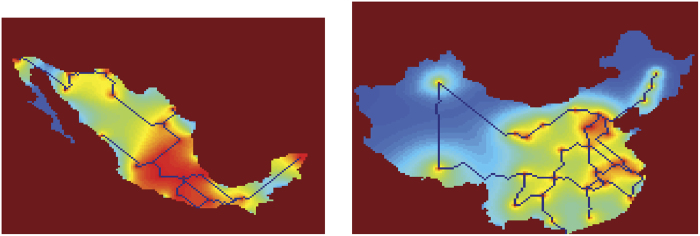
The transportation networks developed by the Physarum-inspired cellular automata models. (**a**) Mexico highways (**b**) China motorways. The maps are generated by the *Physarum*-inspired cellular automata models. Maps created by MAT and GS.

Figure 4(a) shows the Mexico highway networks built by Tsompanas’s model. The numbers near blue circles denote the *α*'s value associated with each formulated network. In terms of the transportation performance, the cost of Mexican highways built by the cellular automata model is least among all the alternative networks, which is greater than the MST by a factor of 1.2. Regarding the network performance, the lower its value is, the better the performance is. However, for the network build by the cellular automata model, its transportation performance is about 0.97, which is the worst in all the networks. For the network built by real *Physarum*, it can be seen that it has a factor of 3.8 when compared with the MST. As for the network formulated by the *Physarum* algorithm, when *α*’s value ranges from 0.01 to 0.26, the transportation performance fluctuates gradually. Among them, the networks have less cost but high transportation performance when *α* ranges from 0.05 to 0.26. This demonstrates that the proposed methods are flexible. In a real-world environment, we can determine the topology of the network and the value *α*according to the specific budget. It can be noted that when *α* changes from 0.05 to 0.26, the *Physarum* algorithm can build networks with higher performance but lower cost when compared with other networks. In summary, the *Physarum* algorithm can achieve better and flexible results with marginally lower costs.

**Figure 5:**
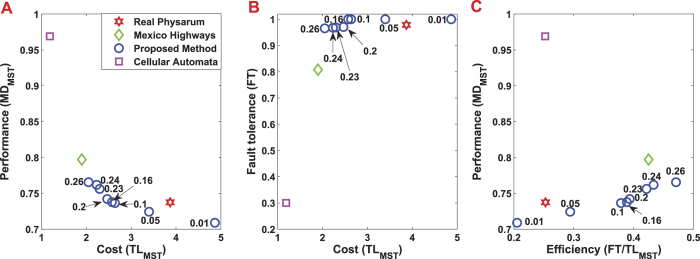
Transport performance, fault tolerance, and efficiency for the network developed by real *Physarum*, the Mexican highways, the network constructed by the *Physarum* algorithm and the network built by the cellular automata model. **(A)** The transportation performance of each network. This index is measured as the minimum distance between all pairs of nodes, then it is normalised to the MST (*MD*_*MST*_). The open green diamond represents the performance of real Mexican highways while the open red hexagram denotes the network developed by real *Physarum*. The open blue circles represent the transportation performance when *α* has different values. The open pink square represents the transportation performance of the network developed by the cellular automata model. **(B)** Fault tolerance (FT), which is measured as the probability of disconnecting parts in the network when a single link is removed. **(C)** The efficiency of each formulated network.

Our *Physarum* algorithm also outperforms other methods in terms of fault tolerance (FT). As can be seen in Fig. 5(B), the network generated by the cellular automate model has the lowest fault tolerance about 0.3, which means about 70% of faults in this network will lead to the disconnection of any part. However, both the network constructed by real *Physarum* and the *Physarum* algorithm have high fault tolerance. When *α* is 0.01, 0.05, 0.1, 0.16, the formulated networks show the best fault tolerance, which is equal to the maximum tolerance to a random failure of a single link. With the increase of *α*, fault tolerance of a network decreases gradually. As for the network grown by real *Physarum*, its fault tolerance is still higher than Mexican highways, which is about 0.9787.

The trade-off between the cost and fault tolerance is measured by *FT/TL*_*MST*_. Mexican highways have a high efficiency, which is about 0.42. For the real *Physarum*, it has a factor of 0.25. As for the networks formulated by the *Physarum* algorithm, the efficiency factor becomes the highest when *α* is 0.26. When *α* has different values (in other words, when the cost is different), the *Physarum* takes different measures to adapt to these environments spontaneously. For the cellular automate-based network, the corresponding value is 0.25, which is the third lowest value for the efficiency indicator among all the alternative models.

### Application to China Motorways Network

**Figure 6:**
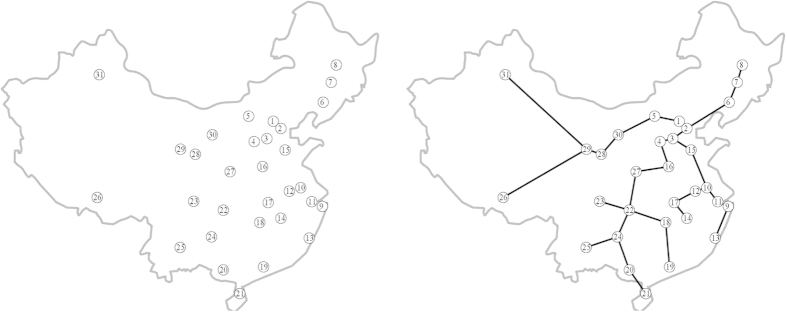
Basis of experiments with China^66^. (**a**) Configuration of sources of food representing major urban areas. (**b**) Minimum spanning tree. The maps are generated using the locations of these cities and the boundaries of China. Maps created by AA.

In China motorways network, we choose 31 most populated major urban areas approximately corresponding to distribution of population densities by 2010^55^ and they are shown in Fig. 6(a). Figure. 6(b) shows the minimum spanning tree of China motorways network. Table 2 displays the population and economic power of each city.

**Figure 7:**
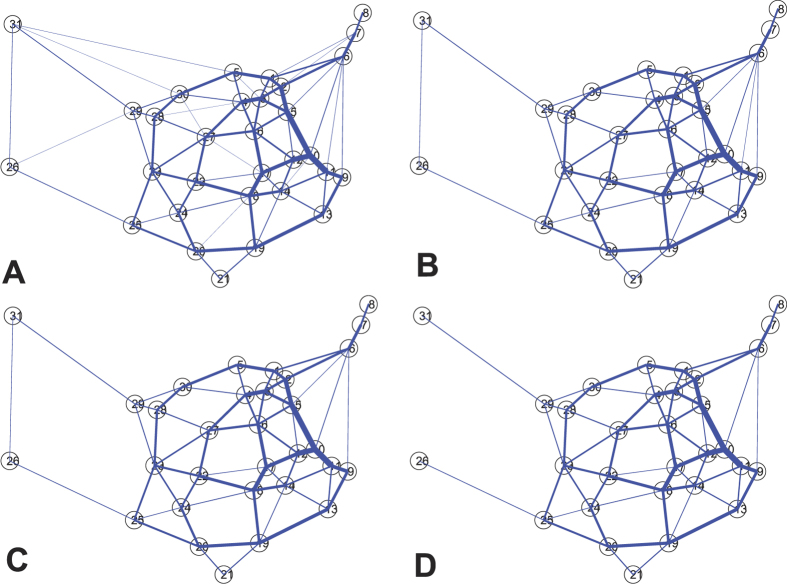
Comparison of the networks constructed by the *Physarum* for different values of α. (**A**) The network constructed by *Physarum* when the edges with conductivity *α* less than 0.02 are filtered out. (**B**) The network with edges with *α* less than 0.05 are eliminated. (**C**) The constructed network when *α* is 0.07. (**D**) The developed network when *α* is 0.09.

Similarly, we can develop the networks by filtering out the edges with conductivity less than *α*. Figure 7 shows us the networks for *α* is 0.02, 0.05, 0.07, and 0.09, respectively. As can be seen in Fig. 7, with the increase of *α*, some unimportant edges gradually disappear while the critical links are retained. The thickness of every edge reflects the actual size of the traffic flow between different cities. It can be seen that most of the edges with bigger traffic flow are mainly distributed in the central and southeastern China.

**Figure 8:**
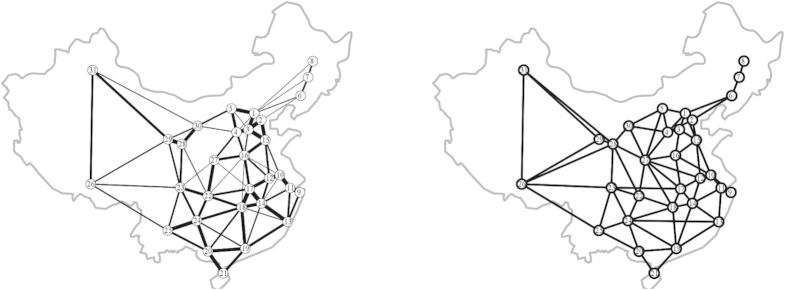
Transport networks. (**a**) Developed by the slime mould. (**b**) Real China motorways. The maps are generated using the networks developed the slime mould and real China motorways, respectively. Maps created by AA.

**Figure 9:**
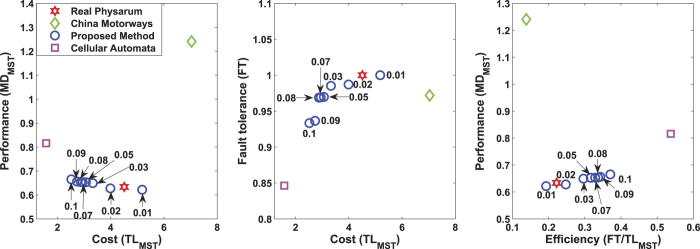
Transport performance, fault tolerance, and efficiency for the network developed by real *Physarum*, the China motorways, the network constructed by the *Physarum* algorithm and the network built by the cellular automata model. **(A)** The transportation performance of each network. The open green diamond represents the performance of real China motorways while the open red hexagram means that of the network developed by real *Physarum*. The open blue circles represent the transportation performance for different values of *α*. The open pink square represents the transportation performance of the network developed by the cellular automata model. **(B)** Fault tolerance (FT) **(C)** The efficiency of each formulated network.

Figure 8 shows the slime mould approximation of transport network in China and the real motorways graph, respectively. Let us compare the transport performance, fault tolerance, and the efficiency of each network. The motorway network shown in Fig. 9(A) has highest cost and lowest performance. China motorways cost is bigger than MST by a factor of 7. The minimum distance between all pairs of nodes in the motorway network is higher than MST by about 25% percent. Both the networks developed by real *Physarum* and the proposed *Physarum* algorithm have less cost. When *α* is 0.01, *Physarum* algorithm has more cost when compared with the network built by real *Physarum* but its performance is better than the performance of the real *Physarum*. In addition, with the increase of *α*, the performance of every network constructed by *Physarum* algorithm decreases gradually while its cost reduces by a larger size. For the cellular automata based network, the constructed network has lowest cost but the performance is the second worst in all the alternatives.

As for the fault tolerance of each network, from Fig. 9, it can be noted that although the cost of China motorways is very high, its fault tolerance is still lower when compared with the network formulated by real *Physarum* and the network built by *Physarum* algorithm when *α* = 0.01. With the change of *α*, the fault tolerance of the networks built by *Physarum* algorithm changes a little when compared with the change of its cost. While the network formulated by the cellular automate model has the lowest cost, its fault tolerance is also lowest among all alternatives. The fault tolerance requires a presence of redundant links in the network therefore it increases the cost of the network.

Finally, let us focus on the efficiency of every network. For *α* = 0.1 the network has the highest efficiency. When *α* changes, the cost’s change amplitude is greater than the fault tolerance’s. Among these networks, China motorways has the lowest efficiency while the network formulated by real *Physarum* is the third lowest. Although the network formulated by the cellular automata model has the highest efficiency, both the performance and the fault tolerance of this network is very worse. In the real-world transportation systems, we need to account for all the three parameters. From this point of view, all the above may suggest that the networks formulated by *Physarum* algorithm are highly efficient. Thus, it is useful to implement this method into real-world applications, such as the transportation network design.

## Discussion

We applied a model of foraging behaviour of slime mould *Physarum polycephalum* to solve a network design problem by maximising transport capacity of the network and minimising the size and length of the network. The *Physarum* algorithm solved the network design problem by developing competition between transport routes: the links with high transportation loads increase their conductivity while less used links are removed. We demonstrated the efficiency of the proposed algorithm by comparing networks produced by the *Physarum* algorithm with networks of man-made highway network in Mexico and motorways networks in China and protoplasmic transport networks grown by the slime mould on a map of major urban areas of Mexico and China and a *Physarum*-inspired cellular automate model. The networks were compared in terms of costs, fault tolerance and efficiency. We demonstrated that the *Physarum* algorithm produces network which are superior in terms of costs, tolerance and efficiency.

Further research will develop in two directions. First, we will adapt the algorithm to the design of sensor, mobile and telecommunication networks. One possible extension of the algorithms would be to incorporate traffic congestion into the network design problem or to consider the problem with traffic equilibrium constraint. Second, we will explore a possibility of implementing the algorithm on a parallel computer. The slime mould is an intrinsically parallel computer: it senses its environment via thousands of receptors distributed in its body, it makes ‘calculations’ via interactions of excitation and peristaltic waves originated from thousands of bio-chemical oscillators. Thus most algorithms inspired by the slime mould are receptive to parallelisation. Ideally we can ‘physically’ map networks optimised into a parallel processor: each elementary processor will be ‘responsible’ for a single node of the network. Figuratively speaking, nodes of the network will be interacting with each other and collectively evolving to an optimal topology of the network.

## Methods

The proposed method consists of two steps. First, we analyse the traffic flows in a network based on the gravity model. Second, the *Physarum* algorithm is employed to deal with the network design problem.

### Network Design Problem^56^

A highway network can be described in terms of nodes or vertices, connected by links. Some of the nodes represent the origins of the transportation demand while others are the destinations of the traffic flow. The network design problem (NDP) is to select links in a network to satisfy the demands of transport capacity and minimise overall costs of transportation^56^.

Consider a network *G*(*V,E*), where *V* denotes a set of nodes, a weight function *L*, a budget *B* and a criteria threshold value *C*. Is there a subgraph *G*′(*V,E*′) of *G* with weight and criterion value *F*(*G*′)≤*C*, where *F*(*G*′) denotes the sum of the weights of the shortest paths in *G*′ between all pairs of vertices?

### *Physarum Polycephalum* Inspired Shortest Path Finding Model

*Physarum Polycephalum* is a large, single-celled amoeboid organism forming a dynamic tubular network connecting the discovered food sources during foraging. The mechanism of tube formation can be described as follows. Tubes thicken in a given direction when shuttle streaming of the protoplasm persists in that direction for a certain time. There is a positive feedback between flux and tube thickness, as the conductance of the sol is greater in a thicker channel. With this mechanism in mind, a mathematical model illustrating the shortest path finding has been constructed^49^.

Suppose the shape of the network formed by the *Physarum* is represented by a graph, in which a plasmodial tube refers to an edge of the graph and a junction between tubes refers to a node. Two special nodes labeled as *N*_1_, *N*_2_ act as the starting node and ending node, respectively. The other nodes are labeled as *N*_*3*_, *N*_*4*_, *N*_*5*_, *N*_*6*_, etc. The edge between node *N*_*i*_ and *N*_*j*_ is expressed as *M*_*ij*_. The parameter *Q*_*ij*_ denotes the flux through tube *M*_*ij*_ from node *N*_*i*_ to *N*_*j*_. Assume the flow along the tube be an approximately Poiseuille flow, then flux *Q*_*ij*_ can be expressed as:


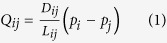


where *p*_*i*_ is a pressure at a node *N*_*i*_, *D*_*ij*_ is a conductivity of a tube *M*_*ij*_, and *L*_*ij*_ is its length.

By considering that the inflow and outflow must be balanced, we have:


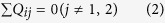


For the source node *N*_1_ and the sink node *N*_2_ the following two equations hold


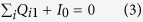



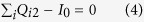


where *I*_0_ is the flux flowing from the source node and *I*_0_ is a constant value here.

In order to describe such an adaptation of tubular thickness we assume that the conductivity *D*_*ij*_ changes over time according to the flux *Q*_*ij*_. An evolution of *D*_*ij*_(*t*) can be described by the following equation:


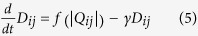


where *γ* is a decay rate of the tube. The equation implies that a conductivity becomes nil if there no flux along the edge. The conductivity increases with the flux. The *f* is monotonically increasing continuous function satisfying *f*(0) = 0.

Then the network Poisson equation for the pressure can be obtained from the Eq. (1)-(4), 2, 3, 4 as follows:


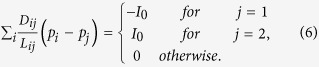


By setting *p*_2_ = 0 as a basic pressure level, all *p*_*i*_ can be determined by solving Eq. (6) and *Q*_*ij*_ can also be obtained.

In this paper, 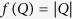 is used because 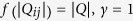, *Physarum* can always converge to the shortest path regardless of whether the distribution of conductivities in the initial state is random or biased^49^. With the flux calculated, the conductivity can be derived, where Eq. (7) is used instead of Eq. (5), adopting the functional form 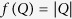.


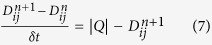


Here, 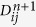 represents the conductivity on link (*i*,*j*) in the n + 1 iteration. The first part |*Q*| in the above equation means the acquired energy while the second part 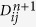 denotes the energy consumed by *Physarum*. For details, please refer to Ref. 49.

### The Gravity Model

Gravity models are trip distribution models, which have been widely used in transportation systems for estimating the traffic flow between the origins and the destinations^57^,^58^,^59^,^60^,^61^,^62^. The gravity model adapts the concept of the law of universal gravitation: it takes into consideration the population of two different places, corresponding to mass in gravity, and the distance between them. The gravity model can be expressed in the following form:


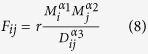


where *F*_*ij*_ represents the traffic flow starting from node *i* to node *j*; *M*_*i*_ and *M*_*j*_ denote the economic sizes of these two places, respectively; *D*_*ij*_ is an economic cost associated with these two positions, such as the distance between them, *G* is an index and it has a constant value. Here, the traffic flow for an individual city is meant to the sum of outward traffic flow.

In the past decades, many researchers have shown that the traffic flow is highly dependent on Gross Domestic Product (GDP) of the associated areas. For example, Cline *et al.*^63^ demonstrated that there was a positive relationship between GDP and freight traffic. Zhang and Guo^64^ found that the air traffic flow of Beijing International Airport and its corresponding GDP was positively correlated with the correlation coefficient up to 0.968. From this point of view, GDP can be used to predict the actual traffic flow. Except that, GDP is a comprehensive indicator. In this indicator, it has accounted for many factors, such as population, industries, income, etc, which in turn reflects development level of a city. As a result, it is more comprehensive in comparison with population. Hence, we will use GDP to represent the economic sizes of the cities.

On the other hand, with the rapid development of transportation networks, including air traffic networks, railways networks, and highway networks, the world has become smaller than before. In this case, the factor distance is not so important as before. For example, Marimoutou *et al.*^65^ explicitly stated that The larger the partner’s GDP, the less will be the distance effect on trade . Kwon and Jung^59^ revealed that the total bus flows between cities depends on only its population size. As a result, we assume that the traffic flow depends on the square root of the product of the GDP of city A and the GDP of city B, but has no relation with the distance between them. As a result, *α*_1_,*α*_2_ are set the same value 0.5. At the same time, as traffic flow has no relation with the distance between these cities, as a result, *α*_3_ is 0.

**Figure 10:**
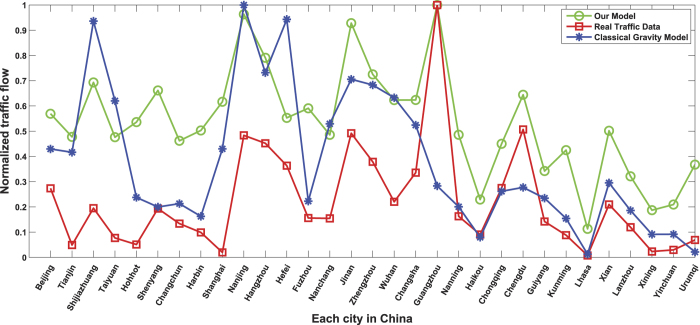
Comparisons between the alternative traffic flow prediction models.

In order to confirm our assumption, we have compared our model with real traffic data and the classical gravity model for the traffic flow of China in 2011. In classical gravity model, they predict the traffic flow as the square root of the product of the population of city A and city B over the square of the distance between them. Based on the data shown in Table 2, by normalizing the traffic flow, we display the traffic flow prediction results between these models in Fig. 10. As can be noted that, the proportions traffic flow trends uncovered in our model and observed in the real traffic data are similar. We can change the prediction results proportionally by adjusting the value of *r* existing in Eq. (8). However, for the classical gravity model, there are obvious differences between the prediction results and the real traffic data. For example, in the classical gravity model, Nanjing has the biggest traffic flow while in real traffic data, Guangzhou has the highest traffic volume. This in turn demonstrates the correctness of our assumption and the efficiency of our method.

### Physarum Model for Network Design Problem

Consider a network *G*(*V*,*E*), where *V* denotes a set of nodes, *E* represents a set of arcs, *L*_*ij*_ represents the length of edge (*i*,*j*). Assume *F* is a set of traffic flow in the network *G* and *F*_*ij*_ denotes the traffic flow from the origin *i* to the destination *j*. *F*_*t*_ represents a number of O-D pairs (Here, the O-D pairs denotes the table of origin-destination demand) in *F*. Here, Eq. (6) is expressed in the following form:


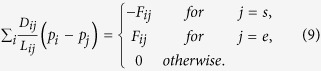


Next, nodes *i* and *j* are starting and ending node in the *Physarum* model, respectively. The *Physarum* algorithm runs only for one iteration when *i* = *j*.

Each link in the network is filled with some flux and their conductivity changes correspondingly. As assume the links obtain some energy from the flux whilst some energy will be also consumed. We employ *Physarum* to simulate the traffic flow onthe link *F*_*ik*_ (*i*,*k*) ∈ *E*, the procedure is similar with that of traffic flow *F*_*ij*_. Energy in the network is limited. Therefore, all the links compete with each other for traffic. Unused links gradually fade and disappear.

At this step, we record the conductivity matrix *D*_*kij*_, which expresses the conductivity matrix when the algorithm of *Physarum* starting from node *i* to node *j* is iterated for *k*_*th*_ times. The conductivity matrix of other O-D pairs in the *k*_*th*_ iteration can be retained and they are expressed as 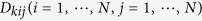. To reflect the functioneach O-D pair plays in the network, the following Eq. (10) is constructed.


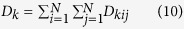


To achieve convergence of the *Physarum* algorithm, we must keep a scale of the conductivity matrix *D* ranging from 0 to 1. As a result, the following normalised measure is obtained:


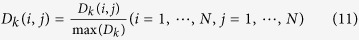


where max(*D*_*k*_) expresses the largest value in the conductivity matrix *D*_*k*_.

In what follows, *D*_*k*_ will be input as the initialised value of the conductivity matrix for the (*k* + 1)_*th*_ iteration. The algorithm runs until a termination criterion is met. There could be several possible termination criteria, including a maximum number of iterations achieved or stationary flux through each tube recorded. In present paper, we adopt the following termination criterion: the algorithm stops when values of conductivity matrix elements stabilize. A general flow of this method is shown in Algorithm 1.

## Additional Information

**How to cite this article**: Zhang, X. *et al.* A Biologically Inspired Network Design Model. *Sci. Rep.* **5**, 10794; doi: 10.1038/srep10794 (2015).

## Supplementary Material

Supplementary Information

## Figures and Tables

**Table 1:** The general data on urban cities, including main towns and states, populations, and economic potentials adopted from^52^.

**Map number**	**Town or capital**	**State**	**Population per state**	**Economic power**
1	Tijuana	Baja California Nore	3122400	8
2	Nogales	Sonora	2499263	12
3	Ciudad Juárez	Chihuahua	3376052	5
4	Hermosillo	Sonora	2499263	12
5	Chihuahua	Chihuahua	3376052	5
6	Nuevo Laredo	Tamaulipas	3174134	11
7	Monterrey	Nuevo Leon	4420909	3
8	Mazatlán	Sinaloa	2650499	15
9	Ciudad Victoria	Tamaulipas	3174134	11
10	San Luis Potosí	San Luis Potosí	2479450	14
11	Guadalajara	Jalisco	6989304	4
12	Léon and Guanajuato	Guanajuato	5033276	9
13	Morelia	Michoacan	3971225	22
14	Edo. México,	*	14739060,	2
	DF, Puebla		8839361,	1
			5624104	7
15	Xalapa, Veracruz	Veracruz	7270413	6
16	Chilpancingo, Acapulco	Guerrero	3143292	18
17	Oaxaca, Huatulco	Oaxaca	3551710	20
18	Tuxtla Gutiérrez	Chiapas	4483866	17
19	Merida and Cancún	Yucata¡än, Qintana Ro	1909965, 1290300	21, 19

*indicates that more than one big city is enumerated on such row

**Table 2:** The general data on urban cities in China, including main town and state, population, and economic power adopted from^55^.

**Map number**	**Town or capital**	**State**	**Population**	**Economic power (Unit: 10^9^ RMB)**	**Actual traffic flow (Unit: 10^4^)**
1	Beijing	Beijing	20190000	16,251.9393	2,019
2	Tianjin	Tianjin	13550000	11,307.2828	1,355
3	Shijiazhuang	Hebei Province	72410000	24,515.7676	7,241
4	Taiyuan	Shanxi Province	35930000	11,237.5555	3,593
5	Hohhot	Inner Mongolian Autonomous Region	24820000	14,359.88	2,482
6	Shenyang	Liaoning Province	43830000	22,226.70	4,383
7	Changchun	Jilin Province	27490000	10,568.83	2,749
8	Harbin	Heilongjiang Province	38430000	12,582.00	3,834
9	Shanghai	Shanghai	23470000	19,195.69	2,347
10	Nanjing	Jiangsu Province	78990000	49,110.27	7,899
11	Hangzhou	Zhejiang Province	54630000	32,318.85	5,463
12	Hefei	Anhui Province	59680000	15,300.65	5,968
13	Fuzhou	Fujian Province	37200000	17,560.18	3,720
14	Nanchang	Jiangxi Province	44880000	11,702.82	4,488
15	Jinan	Shandong Province	96370000	45,361.85	9,637
16	Zhengzhou	Henan Province	93880000	26,931.03	9,388
17	Wuhan	Hubei Province	57580000	19,632.26	5,758
18	Changsha	Hunan Province	65960000	19,669.56	6,596
19	Guangzhou	Guangdong Province	105050000	53,210.28	1,0505
20	Nanning	Guangxi Province	46450000	11,720.87	4,645
21	Haikou	Hainan Province	8770000	2,522.66	877
22	Chongqing	Chongqing	29190000	10,011.37	2,919
23	Chengdu	Sichuan Province	80500000	21,026.68	8,050
24	Guiyang	Guizhou Province	34690000	5,701.84	3,469
25	Kunming	Yunnan Province	46310000	8,893.12	4,631
26	Lhasa	Tibet Autonomous Region	3030000	605.83	303
27	Xian	Shaanxi Province	37430000	12,512.30	3,743
28	Lanzhou	Gansu Province	25640000	5,020.37	2,564
29	Xining	Qinghai Province	5680000	1,670.44	568
30	Yinchuan	Ningxia Hui Autonomous Region	6390000	2,102.21	639
31	Urumqi	Xinjiang Uyghur Autonomous Region	24820000	6,610.05	2,209
