# Supplementary material for: A Biologically Inspired Network Design Model
Source: Sci Rep. 2015 Jun 4;5:10794. doi: 10.1038/srep10794 (PMC4455180; doi:10.1038/srep10794)
Supplement: Supplementary Information [file srep10794-s1.pdf]

A Biologically Inspired Network Design Model (Supplementary Information)

Xiaoge Zhang[a,d], Andrew Adamatzky[b], Felix T.S. Chan[c], Yong Deng[a,d*], Hai Yang[e], Xin-She Yang[f], Michail-Antisthenis I. Tsompanas[g], Georgios Ch. Sirakoulis[g], Sankaran Mahadevan[d*]

[a]School of Computer and Information Science, Southwest University, Chongqing 400715, China; [b]Unconventional Computing Center, University of the West of England, Bristol BS16 1QY, UK; [c]Department of Industrial and Systems Engineering, The Hong Kong Polytechnic University, Hung Hum, Kowloon, Hong Kong; [d]School of Engineering, Vanderbilt University, Nashiville, 37235, USA; [e]Department of Civil and Environmental Engineering, The Hong Kong University of Science and Technology, Clear Water Bay, Kowloon, Hong Kong; [f]School of Science and Technology, Middlesex University, London NW4 4BT, UK; [g]Department of Electrical and Computer Engineering, Democritus University of Thrace, Xanthi 67100, Greece; [*]Correspondence and requests for materials should be addressed to Y. D. (prof.deng@hotmail.com) and S. M. (sankaran.mahadevan@vanderbilt.edu)

Algorithm 1 *Physarum Polycephalum* Algorithm for Network Design Problem (F, L)

// N is a number of nodes, δ has a threshold value.
// L is a $n \times n$ matrix, L_{ij} denotes the length between node i and node j
// F is a $n \times n$ matrix, F_{ij} represents a traffic flow between node i and node j
Construct a traffic flow F according to Eq. (8)

$$F_{ij} = G\frac{M_i^{\alpha_1} M_j^{\alpha_2}}{D_{ij}^{\alpha_3}}$$

$previousD_{ij} \leftarrow 0 \quad (\forall i, j = 1, 2, \ldots, N)$
$originalD_{ij} \leftarrow 0 \quad (\forall i, j = 1, 2, \ldots, N)$
while $\sum_{i=1}^{N} \sum_{j=1}^{N} |currentD_{ij} - originalD_{ij}| \geq \delta$ **do**
  $previousD_{ij} \leftarrow currentD_{ij}$
  $originalD_{ij} \leftarrow currentD_{ij}$
  $currentD_{ij} \leftarrow 0$
  for $i = 1 : N$ **do**
    for $j = 1 : N$ **do**
      $currentD = currentD + PA(i, j, L, previousD, F_{ij})$
    end for
  end for
  According to Eq. (11), the conductivity is normalised.

$$D_k(i,j) = \frac{D_k(i,j)}{\max(D_k)} \quad (i = 1, \cdots, N, j = 1, \cdots, N)$$

end while
Require: s, e, L, D, f
Ensure: D
function Physarum Algorithm PA(s, e, L, D, f)
// s is the starting node, e is the ending node, f is the traffic flow starting from node s to node e
// D is an initialised conductivity matrix
iteration=0
  while *iteration* < 1 **do**
    Calculate a pressure according to Eq. (9)

$$\sum_i \frac{D_{ij}}{L_{ij}}(p_i - p_j) = \begin{cases} +f & for \quad i = s, \\ -f & for \quad i = e, \\ 0 & otherwise. \end{cases}$$

    $Q_{ij} \leftarrow D_{ij} \times (p_i - p_j)/L_{ij}$ // Using Eq. (1)
    $D_{ij} \leftarrow Q_{ij} + D_{ij}$ // Using Eq. (7)
    $iteration \leftarrow iteration + 1$
  end while
  return D
end function
